# Yield of CT pulmonary angiography in the diagnosis of acute pulmonary embolism: short report

**DOI:** 10.1186/s13104-019-4076-8

**Published:** 2019-01-18

**Authors:** Zhongyi Chen, Simon Deblois, Kevin Toporowicz, Irina Boldeanu, Marie-Odile Francoeur, Manel Sadouni, Luigi Lepanto, Carl Chartrand-Lefebvre

**Affiliations:** 1 Department of Radiology, Centre Hospitalier de l’Université de Montréal (CHUM), 1051 Sanguinet Street, Montréal, QC H2X 0C1 Canada; 2 Health Technology Assessment Unit, CHUM, Montréal, QC Canada; 3 Research Centre, CHUM, Montréal, QC Canada

**Keywords:** Pulmonary embolism, Computed tomography, Oversuse, Yield rate

## Abstract

**Objective:**

To assess computed tomography pulmonary angiography (CTPA) positive yield rate for pulmonary embolism (PE) in a Canadian academic tertiary center.

**Results:**

This one-center retrospective cross-sectional study includes from 5565 (model 1) to 5296 (model 2) patients that were evaluated for suspected PE in 2015, among which 1331 (23.9% (model 1) to 25.1% (model 2)) underwent CTPA. Mean age of CTPA patients was 60.2 ± 16.6 years, of which 575 were males (43.2%). Two hundred eleven CTPA examinations were positive for PE, giving a CTPA positive yield rate of 15.9% (95% CI (13.93–17.87)). One hundred and thirteen (8.1%) CTPA were considered indeterminate, and eleven were considered nondiagnostic (0.8%). Among the 211 CTPA positive for PE, 67 (32%) were proximal emboli, 98 (47%) were segmental emboli and 44 (21%%) were subsegmental emboli. In conclusion, in this retrospective study done in a Canadian academic tertiary center, we report a positive rate of 15.9% for PE detection with CTPA, which is above the generally accepted lower threshold of 10% for the yield of CTPA.

## Introduction

In the recent years, the increasing use of pulmonary computed tomography angiography (CTPA) in the evaluation of suspected pulmonary embolism (PE) has raised the issue of overuse. One argument for an overuse of CTPA is a low positive yield rate, i.e. a low positivity rate for PE. Although a definitive ideal positivity rate for CTPA has not been established, a 10% rate is generally accepted as the threshold under which overuse of CTPA should be considered [1, 2]. Another possible concern is the tendency of CTPA to overdiagnose clinically insignificant cases of PE, as evidenced by the lack of improvement in mortality rate of PE after the introduction of CTPA [3]. We would like to report the results of a study assessing CTPA yield for PE done in our medical center, a Canadian academic tertiary center.

## Main text

### Materials and methods

#### Study design

It is a retrospective cross-sectional study that included all patients at the University of Montreal Medical Center (CHUM) suspected of acute PE between January and December 2015 (inclusive). Approval was obtained from the CHUM institutional review board (16.091) and the requirement for informed consent was waived.

#### PE models

Two models were used to retrospectively identify patients being suspected of PE. In model 1, patients were considered as being suspected of PE if they underwent at least one of the following tests: D-dimer dosage, CTPA or ventilation-perfusion scintigraphy. In model 2, the same search criteria were used, except that patients who underwent venous Doppler of lower limb(s) were subtracted from the patients who had D-dimer testing.

#### Data collection

Data were gathered from the CHUM’s electronic medical records system. In the CHUM, CT scanners used at the time of the study were 64- to 256-slice systems. Most CTPA examinations were interpreted by cardiothoracic radiologists. A minority of examinations were interpreted by non-cardiothoracic radiologists, especially off-hours. All on-call interpretations by a radiology resident is reviewed by a staff radiologist, and recorded in the final report.

The following information was gathered from the radiology reports: age, sex, scan result with location of the most proximal embolus if applicable, and relevant clinical data written on the request (hemoptysis, dyspnea, chest pain, syncope, desaturation, signs of right-sided heart failure).

CTPA results from the radiology reports were classified as positive (presence of PE), negative (absence of PE), indeterminate or nondiagnostic. A CTPA was classified as positive for PE when the radiologist’s interpretation showed a definitive diagnosis of PE; clear absence of PE was classified as a negative result; presence of intraluminal images doubtful for PE, due to artefacts, suboptimal opacification or to other causes was classified as indeterminate; a nondiagnostic result was used when no clear information could be drawn from the CT angiogram. For positive CT angiograms, the arterial level (main, lobar, segmental or subsegmental) of the most proximal PE was noted.

#### Statistical analysis

Continuous variables are expressed as mean ± standard deviation (SD), and categorical variables are expressed as count (percentage of total), unless otherwise specified. Descriptive statistics were compiled using spreadsheet software (Microsoft Excel 2013 (Microsoft corp., Redmond, USA)). CT positive yield rate is defined as the quotient of number of positive CT scans/total number of CT scans. Further statistical analyses were done using software (SPSS version 23 (IBM corp., Armonk, USA)). P-values less than 0.05 were considered statistically significant.

### Results

In total, from 5565 (model 1) to 5296 (model 2) patients were evaluated for suspected PE in the CHUM in 2015, among which 1331 (23.9% (model 1) to 25.1% (model 2)) underwent CTPA. Mean age of CTPA patients was 60.2 ± 16.6 years, of which 575 were males (43.2%). Demographics of patients who underwent CTPA are summarized in Table 1. Two hundred eleven CTPA examinations were positive for PE, giving a CTPA positive yield rate of 15.9% (95% CI (13.93–17.87)). One hundred and thirteen (8.1%) CTPA were considered indeterminate, and eleven were considered nondiagnostic (0.8%).

**Table 1:** Demographics and symptoms of patients who underwent CTPA

Number of CTPA scans	1331
Age (years)	60.2 ± 16.6
Male sex (*n*)	575 (43.2)
Hemoptysis (*n*)	610 (45.9)
Dyspnea (*n*)	390 (29.3)
Chest pain (*n*)	417 (31.4)
Syncope (*n*)	340 (25.6)
Desaturation (*n*)	147 (11.1)
Signs of right-sided heart failure (*n*)	16 (1.20)

Numbers in parentheses are percentages. Signs and symptoms were collected from the scan requests

Among the 211 CTPA positive for PE, 67 (32%) were proximal emboli, 98 (47%) were segmental emboli and 44 (21%%) were subsegmental emboli (Table 2).

**Table 2:** Pulmonary emboli by location (N = 211 CTPA positive for PE)

Most proximal location	Number of positive CTPAs
Main pulmonary artery	23 (11.1)
Lobar	44 (21.1)
Segmental	98 (46.9)
Sub-segmental	44 (21.1)

### Discussion

In a recent systematic review done by our group, the baseline diagnostic yield of CTPA for PE was shown to vary from 4.7 to 31% [4]. In 7 (58%) out of the 12 included studies in this review, the diagnostic yield was lower than the generally accepted threshold of 10% [1, 2]. Using this 10% cut-off, 3 of the high-yield studies were from the United States (US), as well as one from Spain and another from Australia. In comparison, all low-yield studies were from the US, raising the possibility that decreased CTPA yields in US medical centers could reflect concern for malpractice litigation, as was suggested by previous authors [2, 5]. Of note, a recent study of Sharma and Lucas from Scotland [6] shows a positive yield of CTPA for PE over 10%, like ours and another study conducted in another academic center in Canada [7].

CTPA provides accurate and readily available information to the ordering physician on the presence or absence of PE, as well as on other chest pathologies. This is why it could be tempting to use CTPA as a one-stop tool for the diagnosis of PE. However, it should be remembered that CTPA has been validated as part of clinical decision rules [8, 9], involving the application of a pre-test clinical assessment (Wells criteria, PERC, Charlotte rule, Geneva score, for example) as well as D-dimer testing when clinically relevant. Moreover, clinical guidelines clearly recommend the use of CTPA as part of these clinical decision rules [10–12]. The choice of a specific rule and D-dimer testing should be based on the prevalence of PE within the clientele’s clinical characteristics and characteristics of the health care setting [13, 14]. Further research is needed to assess the impact of interventions aimed at promoting the use of validated clinical decision algorithms and improving the appropriateness of imaging use in the diagnostic workup of PE.

In conclusion, in this retrospective study done in a Canadian academic tertiary center, we report a positive rate of 15.9% for PE detection with CTPA, which is above the generally accepted lower threshold of 10% for the yield of CTPA.

## Limitations

Our study involves no clinical follow-up data. Second, although strict definitions of positive, negative, indeterminate or nondiagnostic results for PE were used, there is still the possibility of classification bias. Finally, this study was performed in an academic center, involving CTPA interpretation by cardiothoracic radiologists in most cases, and it remains possible that our results would be altered in a different context.

## Abbreviations

CTPA: computed tomography pulmonary angiography
PE: pulmonary embolism
CHUM: University of Montreal Medical Center
SD: standard deviation
